# Unravelling tumble and swirl in a unique water-analogue engine model

**DOI:** 10.1007/s12650-018-0485-3

**Published:** 2018-03-14

**Authors:** Athanasia Kalpakli Vester, Yu Nishio, P. Henrik Alfredsson

**Affiliations:** 1 KTH Mechanics, Osquars Backe 18, 100 44 Stockholm, Sweden; 2 Graduate School of Engineering, Tohoku University, 6-6-01 Aramaki-Aoba, Aoba-ku, Sendai, 980-8579 Japan

**Keywords:** In-cylinder flow, Tumble, Swirl, Stereoscopic particle image velocimetry

## Abstract

**Abstract:**

The in-cylinder flow prior to combustion is considered to be one of the most important aspects controlling the combustion process in an engine. More specifically, the large-scale structures present in the cylinder, so-called tumble and swirl, before compression are believed to play a major role into the mixing and combustion processes. Their development during the intake stroke and their final strength depend mainly (but not only) on the inlet port design. In the present study, the turbulent large-scale structures during the intake stroke are investigated in a unique water-analogue engine where inlet ports and valve timings can easily be configured and tested. The flow field in the cylinder volume is reconstructed through multi-planar stereoscopic particle image velocimetry measurements which reveal a wealth of vortical structures during the stroke’s various phases. The aim of the present paper is to present and show results from a unique setup which can serve as a test bench for optimisation of inlet port designs to obtain a desired vortical pattern in the cylinder after the intake stroke is finished. This setup can simulate the intake stroke in a much more realistic way as compared to a through-flow setup with a fixed valve lift.

**Graphical Abstract:**

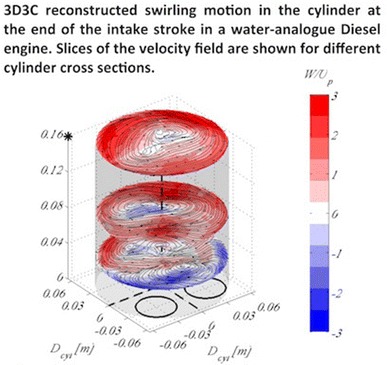

**Electronic supplementary material:**

The online version of this article (10.1007/s12650-018-0485-3) contains supplementary material, which is available to authorized users.

## Introduction

The characteristics of the flow inside the cylinder of internal combustion engines before combustion have long been capturing the interest of engine researchers as well as fluid dynamicists. This is due to the acknowledgement from an early stage that the flow dynamics in the cylinder—both for a spark- or compression-ignition type of engine—is one of the most important factors controlling the combustion process (Borée and Miles 2014; Heywood 1987). Even though it was early recognised by experts in the field that studying the in-cylinder flow dynamics in detail was important, redirecting the engine development from an empirical to a more scientific process is still an ongoing undertaking (Lumley 2001). Nevertheless, it is nowadays largely appreciated that fundamental research of the complex physical phenomena during, e.g. the intake stroke is essential (Bücker 2013; Lumley 2001) and the only way towards optimisation of designs (Towers and Towers 2004).

**Fig. 1:**
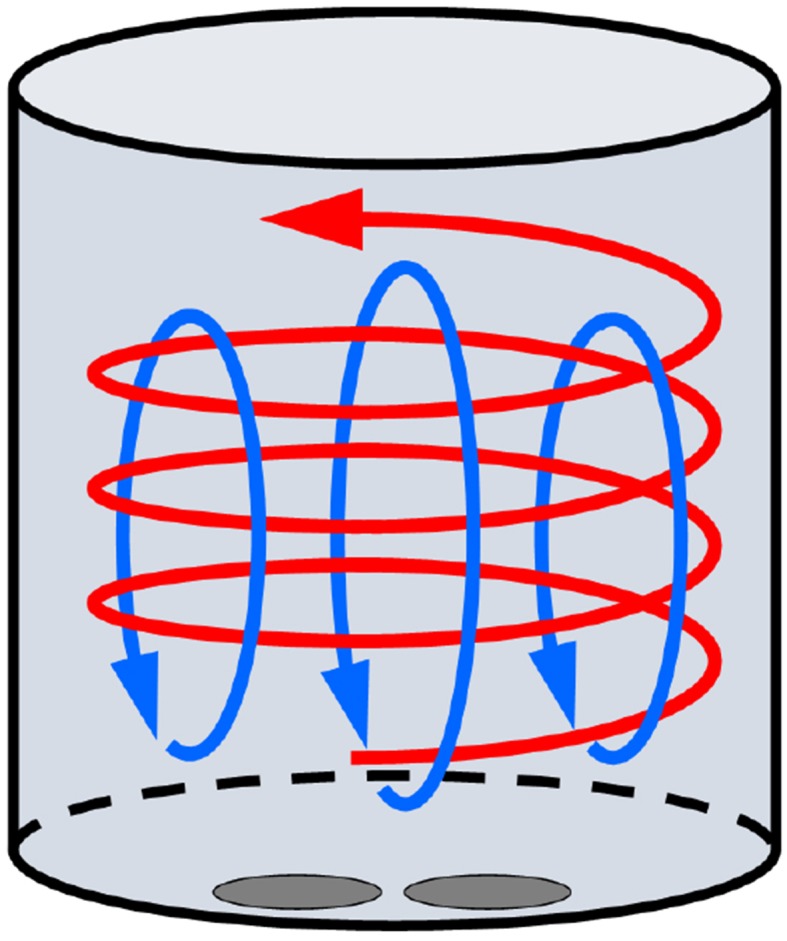
Schematic of the tumble and swirl motions in the cylinder. The red arrow indicates swirl and the blue ones indicate tumble

The air entering the cylinder through the inlet valves forms jets and small-scale turbulence is generated in the shear layers but decays fast. The idea with creating organised, so-called coherent motions, is that some of the momentum of the intake jets becomes encapsulated in these structures and since such large-scale motions dissipate slower than the turbulence they will retain their kinetic energy longer (Lumley 1999). One such motion, so-called swirl, is aligned normal to the cylinder axis and in most cases discussed in the literature it has been quantified by comparisons with solid-body rotation, although this is clearly an oversimplification (Towers and Towers 2004). High swirl is often desired in compression-ignition (CI or Diesel) engines where the flow inside the cylinder controls the air–fuel mixing and burning rates (Heywood 1987). Most CI engines are carefully designed to create a swirling motion to achieve higher mixing rates (for a detailed description on turbulent structures in direct-injection diesel engines, the reader is referred to Ref. Miles (2009). Swirl usually co-exists with another motion, which is mostly associated with spark-ignition (SI) technologies, namely the so-called tumble, which is aligned parallel to the cylinder axis; for a sketch of these motions see Fig. 1.

The strengths of the tumble and swirl motions depend mainly on the design of the port/valve geometries and a description of typical port/valve geometries in SI and CI engines can be found in Ref. Lumley (1999). Tumble and swirl are widely used concepts in in-cylinder flow research nowadays and a common practise is to characterise swirl and tumble within steady-flow engine test benches with equivalent engine geometry and fixed valve positions, see for instance Refs. Mahmood et al. (1996), Pajkovic and Petrovic (2008). Recently, experiments utilising particle image velocimetry to study the in-cylinder flow structures in a steady-flow engine test bench have also been reported (Rabault et al. 2016). Such measurements can be useful to check the swirl or tumble generating capability of the intake ports/valves in a simplified experiment; however, they do not reflect the flow conditions in a real engine since the valves are static and the piston is absent. As noted also by Ref. Söder (2015), with the increasing demand in engine development, deeper knowledge of the large-scale structures during intake, where also dynamic effects are taken into account, is needed.

Turbulence levels have been found to be highest in the early to mid-intake stroke with turbulence production being significantly reduced at the second half of the intake phase as the momentum of the inlet jets decreases (Celik et al. 2001). During the compression stroke, the swirl is conserved, whereas the large-scale tumble motion breaks down into smaller scales. Tumble and/or swirl are associated with more effective combustion, but increasing swirl above some threshold may lead to fuel sprays collision, increasing subsequently the local air/fuel ratio and, therefore, increasing soot emissions and fuel consumption (Jayakumar et al. 2012).

From the above it is made clear that a detailed description and study of swirl and tumble through accurate and advanced methods is necessary for improving the design of the engine towards not only increased efficiency, but also reduced emissions.

Most of the work available regarding in-cylinder flows at real engine conditions are related to gasoline engines due to geometrical and thermodynamical constraints of diesel engines that make optical measurements difficult (Cosadia et al. 2006; Singh et al. 2015). Furthermore, little work, with a couple of exceptions (Borée et al. 2002; Fetter et al. 2000), has been done on generic geometries, i.e. the largest body of literature is dedicated to measuring tumble in specific gasoline engine models (Bücker et al. 2013, 2012; Dannemann et al. 2010; Overbrüggen et al. 2013). Such experiments give valuable results for those specific cases but limit the range of design parameters that can be explored. Designing a generic (and simpler) setup with the aim to investigate different design parameters, e.g. inlet ports, piston geometry, etc. gives more freedom to experiment, but also allows for better resolved boundary conditions and optical access. For example, performing particle image velocimetry measurements in a model square combustion chamber gave the possibility to obtain highly resolved data of the tumbling motion and track the flow structure development (generation, disruption and breakdown) during the intake and compression strokes in detail (Borée et al. 2002).

### An alternative approach

The flow during the intake stroke can be assumed to be incompressible (Khalighi and Huebler 1988) and, therefore, an alternative approach for in-cylinder experiments is to use water as the working fluid in a model engine, which in that case is called “water-analog(ue) engine”. The main advantage using water instead of air is that it allows for better resolved optical measurements, since it allows for refractive index matching to minimise loss of data due to light reflections during visualisation/particle tracking measurements that require the use of powerful lasers. Furthermore, due to the lower velocities it is possible to obtain truly time-resolved and highly spatially resolved data with cameras currently available on the market. However, whenever such a model engine is used it should be ensured that the criteria for similarity between the water-analogue engine and a real engine are fulfilled. This can be done through some simple scaling of the governing parameters, i.e. Reynolds and Strouhal numbers. The Reynolds number is defined as

$$\begin{aligned} Re=\frac{U_\mathrm{p}D\rho }{\mu }, \end{aligned}$$

where ${U_\mathrm{p}}$ is the mean piston velocity, *D* the cylinder diameter and $\rho$ and $\mu$ are the density and dynamic viscosity of the fluid (air or water) medium, respectively. The Strouhal number is defined as

$$\begin{aligned} St= \frac{\omega _p D}{U_\mathrm{p}}, \end{aligned}$$

where $\omega _p$ is the crankshaft angular speed (which can be used to define a suitable time scale for the engine $T_{\mathrm { engine}}\sim 1/\omega _\mathrm{p}$). It turns out that, if the geometric parameters are kept the same between the real and model cylinder, the Strouhal number is also the same (since $U_p\sim \omega _\mathrm{p}$). Then the only parameter that needs to be matched is the Reynolds number, and with the same cylinder dimensions the ratio of piston velocity to the kinematic viscosity should be the same in the model and the engine. For an engine typical pressures can be up to 300 kPa and temperatures 20$^\circ$ above ambient leading to

$$\begin{aligned} \frac{(\rho /\mu )_{\mathrm {engine}}}{(\rho /\mu )_{\mathrm {water}}}\approx 0.2 \end{aligned}$$

A typical mean piston speed is 5 m/s which would then require a piston speed of 1 m/s in the water model, if the water is at room temperature. However, it turns out that the limiting factor for a water model is cavitation that occurs in the flow channel between the valve and the valve seat Koehler et al. (2015) at high velocities. By heating the water used in the model engine (the viscosity thus decreases), it is possible to obtain higher Reynolds numbers, in Refs. Koehler et al. (2015, 2014) some experiments were done at 60$^\circ \mathrm {C}$, thereby decreasing the viscosity by a factor of 2 compared to room temperature. However, it still has not been possible to run a water-analogue engine at equally high Reynolds numbers as in real engines, but it has been shown that at least considering the large-scale structures this lower Reynolds number does not have a great influence (Hess et al. 2012).

One of the pioneering studies using a water-analogue engine to visualise the in-cylinder flow structures was reported by Ref. Khalighi (1991). In that study, shrouded valves were used to create a “pure” tumble motion in the cylinder. It was shown that this tumbling motion was dominating the whole field-of-view and that it was repeatable between the different experimental runs. Visualisations of that tumbling motion were obtained for different engine speeds by means of particle tracking velocimetry.

Scanning time-resolved PIV were performed in a four-valve water-analogue engine and was reported in Refs. Hess et al. (2012); Koehler et al. (2015). Results obtained when using a water–glycerin mixture as the working fluid were compared to pure water results (Hess et al. 2012) and a comparable behaviour of the different fluids was shown, even if the Reynolds numbers did not match in the two cases. The use of water–glycerin as the working fluid helped to avoid distortions from refractive index differences between the fluid and the cylinder wall and it allowed highly resolved volumetric data to be obtained. The results showed a shift of the tumble vortex towards one side of the cylinder and a deformation of the tumble vortex centreline. Horseshoe-type vortices were found to form at the valves generating a strong swirling motion in the early intake phase in a follow-up work (Koehler et al. 2014). Those vortices were additionally believed by the authors to play an important role in cycle-to-cycle variations.

It has finally been appreciated after many years of research that volumetric measurements or 3D simulations are essential to fully understand the structures present in the cylinder (Freudenhammer et al. 2014; Lee et al. 2000; Konrath et al. 2002). However, many of the experimental techniques providing the full flow field still have limitations. For example, using tomographic-PIV, the results are limited to a small volume (Baum et al. 2013), whereas magnetic resonance velocimetry suffers from low spatial resolution (Freudenhammer et al. 2014) and holographic PIV is difficult to set up and implement (Konrath et al. 2002; Overbrüggen et al. 2013).

### Motivation

From all the above, it is made clear that there is need for new detailed studies of the flow dynamics during the intake stroke, since this is when the desired large-scale structures form. Such studies should include various inlet port designs in a setup where also the motion of the valves and piston are simulated. Most works available up to now consider gasoline engines, i.e. few studies investigate both swirl and tumble but rather focus only on tumble.

In the present study, tumble and swirl motions in an engine cylinder are investigated in a specially designed and unique water-analogue engine, i.e. an engine cylinder where water replaces the air during the intake stroke and the piston/valve lift timings can be configured. The three-dimensional and time-dependent flow field is obtained through multi-planar stereo particle image velocimetry (SPIV) measurements. Since the flow during the intake stroke in an engine is nearly incompressible, the flow dynamics are not affected using water as the fluid medium, as long as the non-dimensional parameters including the geometrical characteristics are kept the same between the model and the engine cylinder. This is the first study—to the authors’ knowledge—where a generic water-analogue setup is established and used to reconstruct the full three-dimensional velocity field in the cylinder during the entire intake stroke.

## Experimental setup

**Fig. 2:**
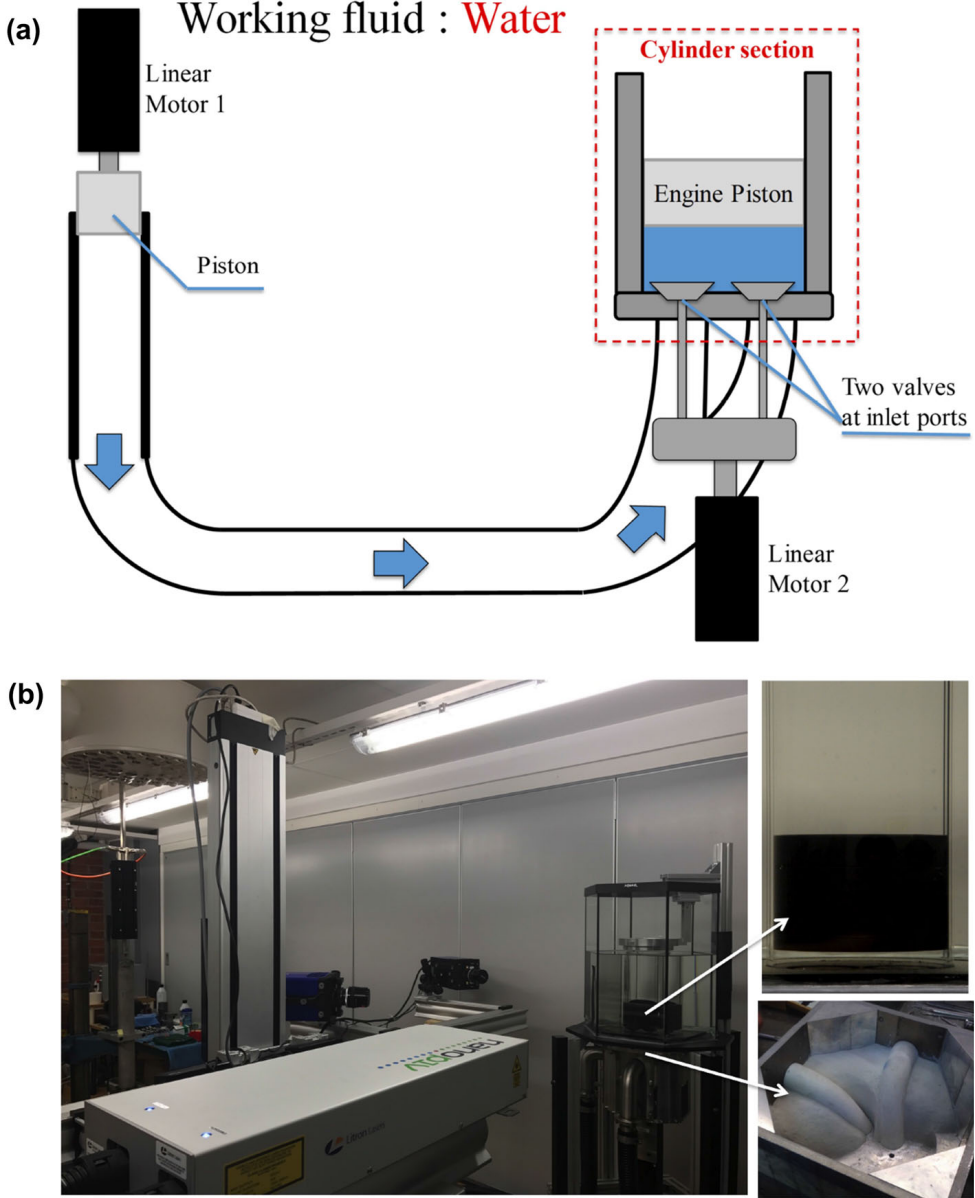
**a** Schematic of the water-analogue engine model. Linear Motor 1 pushes an elongated piston downwards forcing the water (blue arrows) through a hose, which is connected to the inlet of the cylinder head. The engine piston is floating in the cylinder and is therefore forced upwards. The intake valves are connected to Linear Motor 2, which is synchronised with Linear Motor 1. **b** Photograph of the apparatus showing also the SPIV setup. The top insert shows the flat piston floating in the cylinder at the beginning of the stroke, whereas the bottom insert shows one half of the cylinder head before assembling. White arrows indicate the position of those two parts in the apparatus

The experimental setup consists of a unique water-analogue engine cylinder that has been designed such that the cylinder head can easily be interchanged and the piston/valve lift timings can be configured. The model engine cylinder in this study has the same size as a typical heavy-duty truck diesel engine, with a bore of 130 mm and a stroke of 160 mm. Figure 2a shows a schematic of the working principle of the engine model, whereas Fig. 2b shows the setup including the SPIV arrangement as well as the cylinder head geometry used for this study and the flat simplified piston floating in the cylinder. The lower right photograph shows the cylinder head under construction and the two curved pipes seen are the inlet channels (ports) which were designed to create high swirl in the cylinder. The cylinder head is made into two parts (the lower one is seen in the figure) and after moulding the upper part in epoxy the pipes which are made in a different material were removed and two parts were combined to form the cylinder head.

**Fig. 3:**
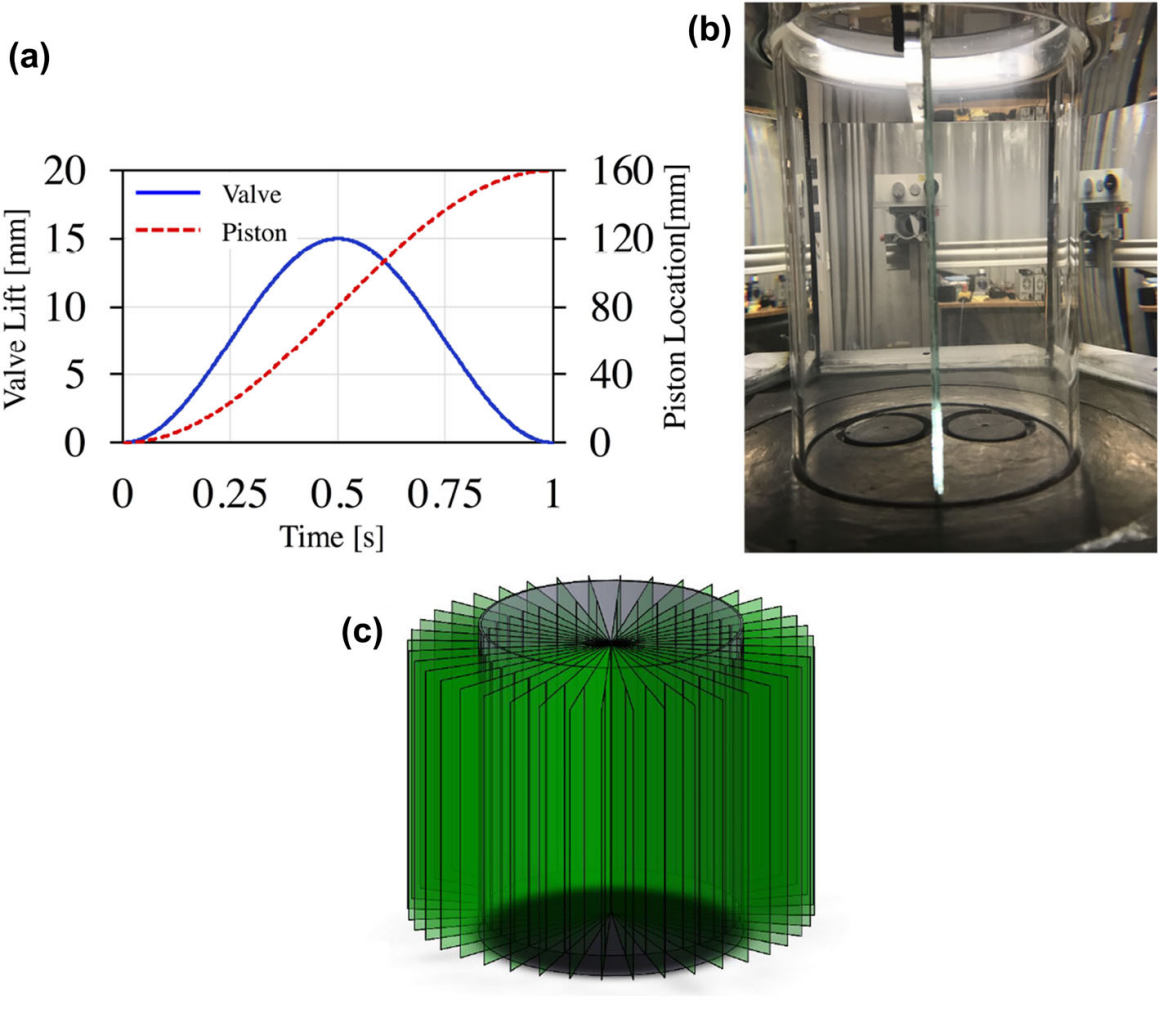
**a** Timing profiles for the piston (red dashed line) and valves (blue line). **b** Plane defined at 0$^\circ$, i.e. a plane located between the two valves. The calibration plate is positioned between the valves cutting the cylinder axis. **c** Illustration of the 3D3C reconstruction procedure, showing the acquired planes used to obtain the whole cylinder volume

The apparatus is of generic nature, i.e. the main setup is made in such a way that parts from real engines such as cylinder heads and valves can be interchanged. The flow is driven by a linear motor, shown in Fig. 2a, which forces the water through the system and pushes the floating piston in the cylinder upwards. The position of the linear motor that controls the water flow is set in the software controlling the linear motor (LinMot Talk 6), in such a way so as to achieve the position profile for the floating piston in the cylinder as it is shown in red in Fig. 3a. It should be noted at this point that to minimise leakage from the system the inlet port is turned “upside-down” compared to a real engine. Therefore, the piston is moving upwards instead of downwards and the bottom dead centre (BDC) in such case is when the piston is at the highest position ($H_\mathrm{cyl}=0.16$ m). The intake valves are moved by a fast-acting linear motor where the lift-opening profile can be controlled and the two linear motors are synchronised so that a real engine intake stroke, both in terms of flow rate and piston/valves motion, can be mimicked. Both linear motors are provided by LinMot®. Figure 3a shows the timing diagram for the valves and the piston chosen for the results shown in this study. The piston used for the results shown here has a flat surface. Furthermore, there is a hexagonal tank surrounding the cylinder, see Fig. 2b, filled with water for refractive index matching. Each of the cameras line of view is perpendicular to one of the surfaces of the hexagonal tank.

**Fig. 4:**
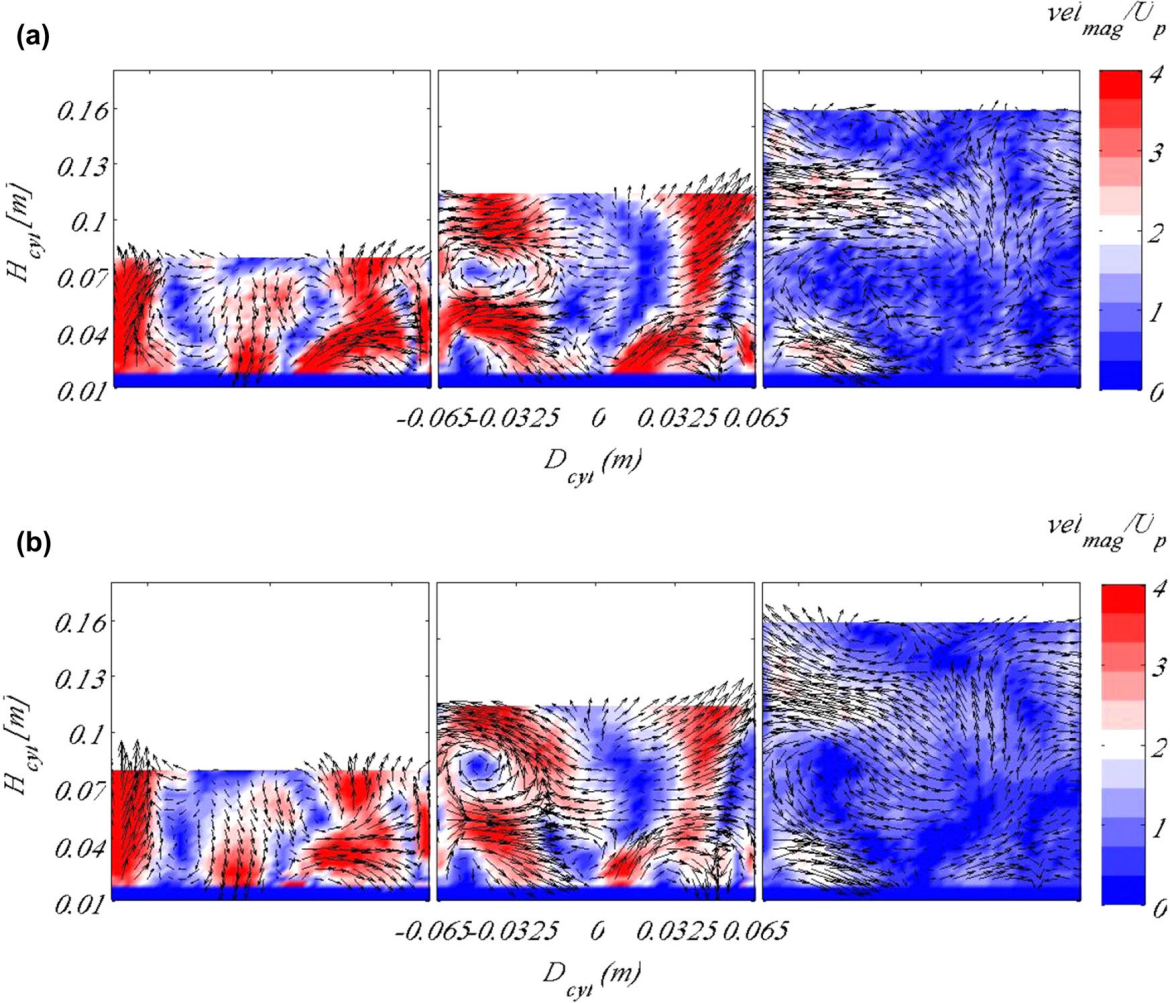
Flow field at three different times during the filling of the cylinder for two different Reynolds numbers and at a fixed valve lift of 10 mm. **a** $Re=10{,}000$ and **b** $Re=26{,}000$. The background contour map indicates the in-plane velocity magnitude, whereas the vectors shows the in-plane velocities. The data were taken at an arbitrary plane

The cylinder head can be rotated to be able to obtain SPIV data at multiple tumble planes, without the need of moving or recalibrating the PIV cameras, allowing a 3D3C reconstruction of the flow field. In Fig. 3b the plane defined at 0$^\circ$ is shown. In total, 21 consecutive tumble planes with 9$^\circ$ step between them are obtained as shown in Fig. 3c. At the end, a linear interpolation between the consecutive planes is performed to obtain the whole cylinder volume.

The SPIV system is from LaVision GmbH and consists of two high-speed cameras (Imager Pro HS 4M, $2016\times 2016$ px resolution) and a Litron Nd:Yag laser (maximum repetition rate 50 Hz). For the seeding of the flow, Vestosint®  particles with a diameter of 56 $\upmu$m were mixed with the water. The SPIV data were taken at a sampling frequency of 50 Hz, whereas 50 cycles were obtained for every plane to obtain a well-converged phase-averaged flow field. The number of cycles for the phase averaging was decided after calculating the correlation coefficient between phase averaged quantities (e.g. such as the vertical velocity) for an increasing number of acquired cycles. A total number of 2500 pairs of particle images were obtained at each plane and the processing of the particle images was performed using DaVis 8.3 by LaVision GmbH, whereas any type of data analysis on the vector fields was done using MATLAB®. The vector fields were calculated through a multi-pass correlation iteration procedure for increased spatial resolution starting from a $64\times 64$ px and decreasing to $16\times 16$ px interrogation area with 50% overlap.

The Reynolds number based on the mean piston velocity and the cylinder bore is $Re=26{,}000$. It is smaller than in a typical diesel engine due to the fact that cavitation occurred at the inlet port at higher velocities. However, the Reynolds number was found to have almost no effect on the large-scale structures, which are of interest in this study. Figure 4 shows the flow field at an arbitrary plane at a fixed valve lift of 10 mm and for two Reynolds numbers, $Re=10{,}000$ and $Re=26{,}000$ for three time instances during the intake stroke. As observed, the existing large-scale vortical structures between the two cases are quite similar. Furthermore, to avoid cavitation the valves had to be covered with water at the beginning of the stroke up to approximately their maximum lift, i.e. 15 mm.

## Results and discussion

In the following section we show results both in the tumble and swirl planes. The primary aim is to show how the setup and reconstruction of the whole cylinder volume works to visualise the flow. However, we also discuss the flow physics in both the tumble and swirl planes and show the differences between fully open stationary valves and moving valves typical for a real engine. Note that all velocity data were acquired in the tumble plane for 21 different (separated by 9$^\circ$) angles and those data are used for the reconstruction of the swirling motion.

**Fig. 5:**
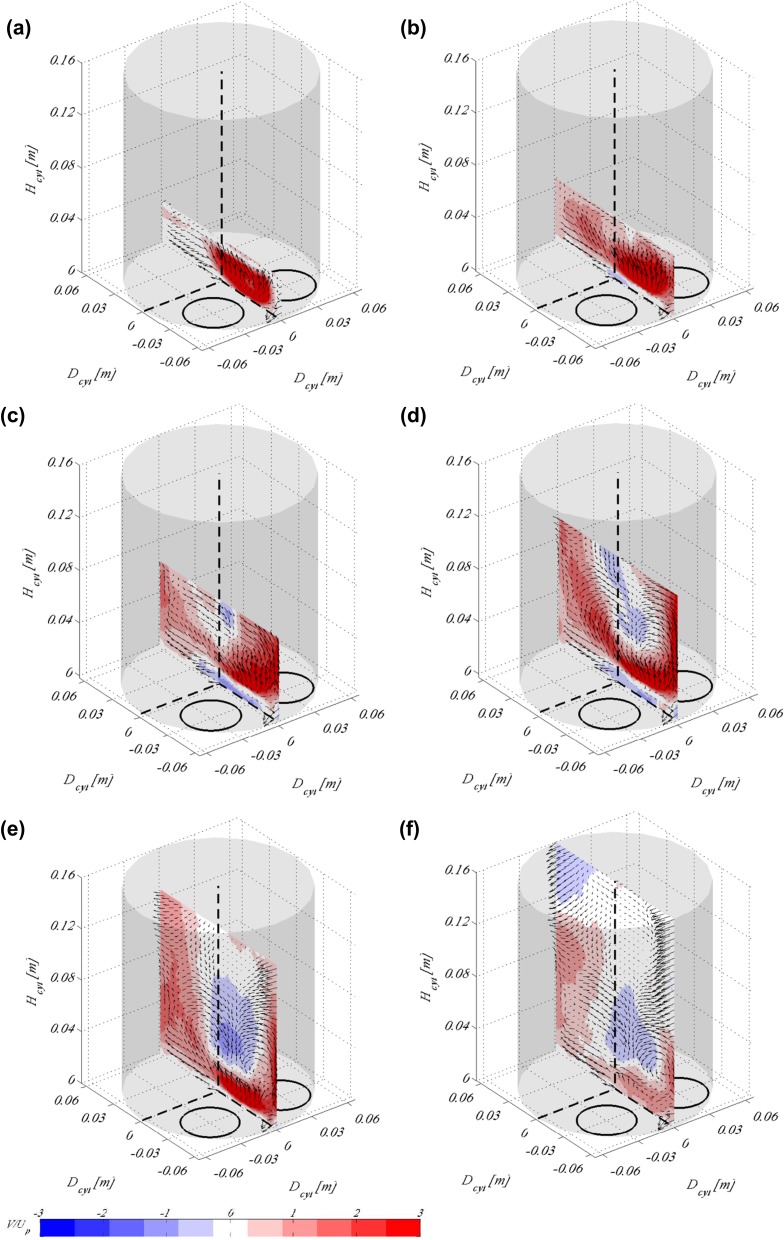
Flow fields showing the tumble plane at 0$^\circ$ of valve rotation for piston location **a** $H_{cyl}=0.032$ m, **b** $H_{cyl}=0.048$ m, **c** $H_{cyl}=0.064$ m, **d** $H_{cyl}=0.096$ m, **e** $H_{cyl}=0.128$ m and **f** $H_{cyl}=0.16$ m. Background contour map indicates the vertical velocity scaled by the mean piston speed, whereas vectors show the in-plane velocity. Results here are shown for moving valves according to the timing diagram in Fig. 3a

Figure 5 shows the evolution of the vortical structures as well as magnitude of the vertical velocity component during the intake stroke for the tumble plane defined as 0$^\circ$, i.e the plane centred between the valves. The data shown here are phase-averaged over 50 consecutive cycles. A bulk tumbling motion is obvious from the figures being created by the inlet jets which are distinct in Fig. 5a. In this plane one mainly observes the influence of the jet from the right valve in the figure, since the cylinder head is designed to create a counter-clockwise swirl (note, if the inlet port is positioned upside-down, as in the present setup). It is observed that the jet has decreased its velocity by a factor of more than 2 from the beginning of the stroke to its end, shown in Fig. 5f, reaching speeds more than three times higher than the mean piston speed at the beginning of the stroke. This is expected since Fig. 5a corresponds to $t=0.2$ s in the timing diagram shown in Fig. 3a and a valve lift of approximately 5 mm. Therefore, the jet is entering the cylinder through a small exit area. At the beginning of the stroke until approximately the first quarter of the stroke (Fig. 5a) the jet occupies the whole plane and there are no vortical structures observed in the flow field. There is fluid flowing only in one direction (as apparent from the colour code). As the valves open up, flow at higher velocity in the opposite direction, i.e. downwards, exists almost at the centre of the cylinder and a vortical structure starts to form at the cylinder wall, as the jet hits it and the local velocity decreases, Fig. 5c, d. That vortex takes a well-defined shape after the second half of the stroke and retains its form until the end of the stroke, Fig. 5f.

**Fig. 6:**
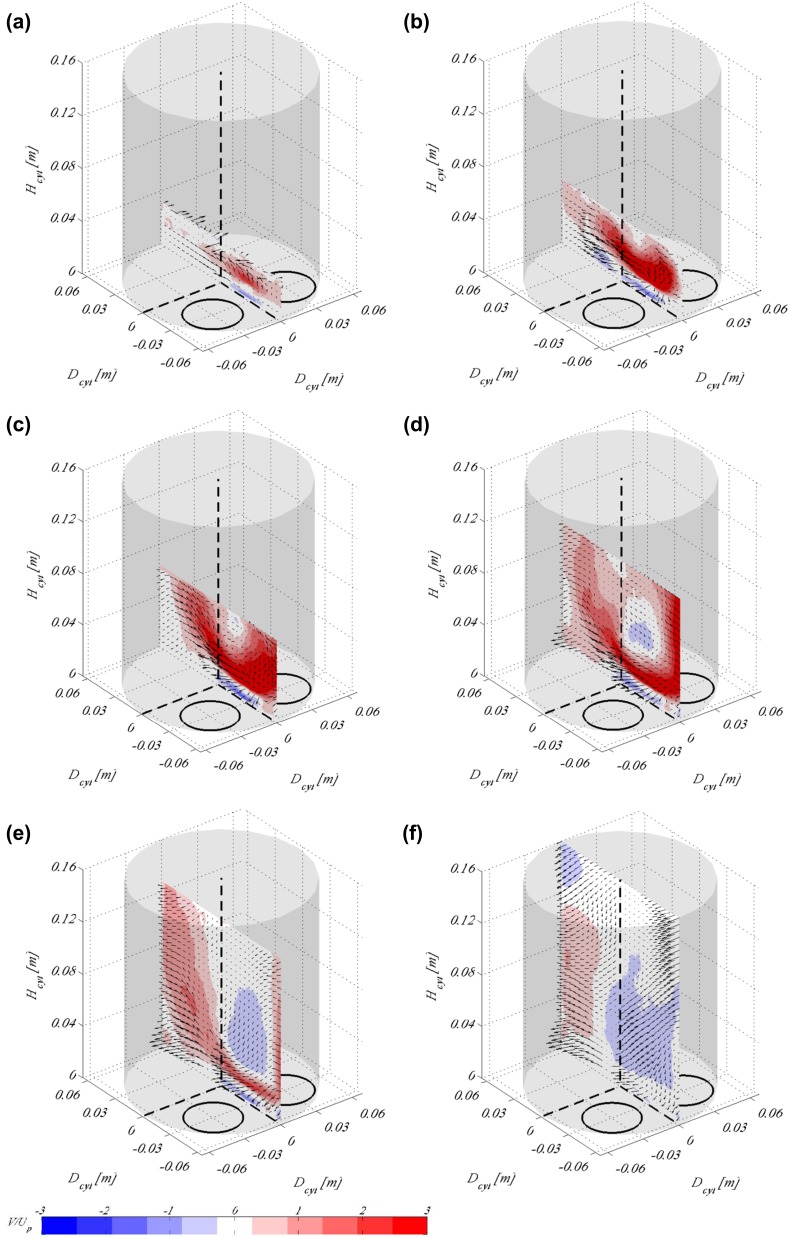
Flow fields showing the tumble plane at 0$^\circ$ of valve rotation for piston location **a** $H_{cyl}=0.032$ m, **b** $H_{cyl}=0.048$ m, **c** $H_{cyl}=0.064$ m, **d** $H_{cyl}=0.096$ m, **e** $H_{cyl}=0.128$ m and **f** $H_{cyl}=0.16$ m. Background contour map indicates the vertical velocity scaled by the mean piston speed, whereas vectors show the in-plane velocity. Results here are shown for fixed valves for a valve lift of 15 mm

In Fig. 6, the same phases are plotted as in Fig. 5 but for a fixed valve lift equal to 15 mm. The differences between the fixed and the moving valve cases are not as significant as one would expect; however, it is observed that at the beginning of the stroke, the inlet jets are not as strong for the fixed valve lift case as it is for the moving valves. Comparing the time instance when the moving valves have reached a lift of 15 mm, it is clearly observed that the vortex close to the cylinder wall that was formed when the valves are moving is not present with fixed valves.

**Fig. 7:**
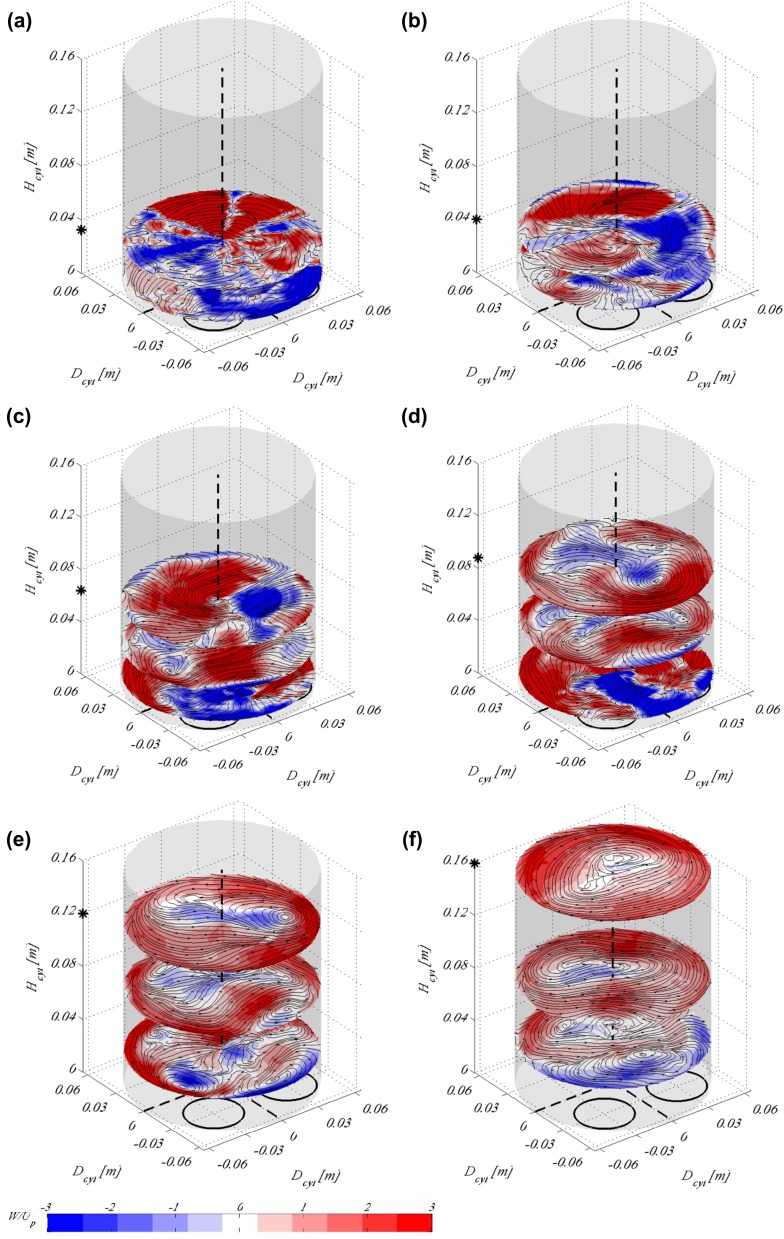
3D3C reconstruction of the swirling motion from the SPIV measurements taken at 21 planes, shown for various cylinder heights and for four piston locations during the intake stroke. Dashed black lines indicate the origin of the cylinder (passing through the centre), whereas the two circles indicate the position of the valves at 0$^\circ$. Contours indicate the azimuthal velocity scaled by the mean piston speed, whereas streamlines are the in-plane velocity. The piston is positioned at **a** $H_{cyl}=0.032$ m, **b** $H_{cyl}=0.048$ m, **c** $H_{cyl}=0.064$ m, **d** $H_{cyl}=0.096$ m, **e** $H_{cyl}=0.128$ m and **f** $H_{cyl}=0.16$ m. The piston position is also indicated by a black asterisk on the $z-$axis. Results here are shown for moving valves according to the timing diagram in Fig. 3a. A movie of subfigure **f** is supplemented as an Online Resource

Figure 7 shows the swirling motion reconstructed from the SPIV data taken at the 21 tumble planes around the cylinder. Cylinder cross sections are visualised for the same physical piston positions, (i.e. time instances in the timing diagram) as in Fig. 5. It is obvious that the 3D3C reconstruction works well and provides a good presentation of the out-of-plane motion in the cylinder. At the beginning of the stroke (Fig. 7a, b) when the inlet jets are strong (refer to previous image), there is hardly any vortical structures observed in this plane. It is first at the end of the first half of the stroke (Fig. 7c) that two, almost symmetrical, vortices start to form in the flow field. A clear single vortex spanning the whole cylinder volume with its core close to one of the cylinder walls appears after the valves open fully and the jets start to decrease in strength, see Fig. 7d. In the second half of the stroke, when the valves have reached their highest lift and start closing again, this swirl takes successively a clearer shape with its core moving from the cylinder walls towards its centre. At the end of the stroke, a well-defined swirl is visible rotating counter-clockwise, Fig. 7f.

As already mentioned in Sect. 2 the inlet port used in this study is one that is designed in such a way as to create high swirl. As apparent from Fig. 2b, where the inlet port is shown during manufacturing, the cylinder head consists of two inlet pipe bends positioned in such a way as to form a non-symmetric inlet port geometry. It is, therefore, expected that as the inlet jets enter the cylinder through such a configuration they will create a swirling motion with its centre off the cylinder axis. Since the inlet jets are strong in the beginning of the cycle, as apparent from Fig. 5, well-established large-scale structures such as the tumble and swirl vortices are formed and remain strong at the end of the stroke.

## Conclusions

The in-cylinder flow during the intake stroke before compression is studied in detail using a unique water-analogue model. The uniqueness of the present setup is its universality, parts can easily be exchanged so that various inlet port designs can be tested and optimised. The complicated flow field of the interaction between two large-scale motions acting in two different planes is easily captured using multi-planar stereoscopic particle image velocimetry and by rotating the inlet port instead of the PIV system. In such a way the whole cylinder volume is captured through a 3D3C reconstruction procedure. Results shown in the present work present the possibilities that this technique has as well as how the phase-averaged flow field evolves and how the various turbulent structures interact with each other throughout the intake stroke. This is the first time that such a versatile experimental setup is being exploited for the study of swirl and tumble in a model engine and the flow field is captured for the whole cylinder volume with high temporal and spatial resolution.

## Electronic supplementary material

Below is the link to the electronic supplementary material.

Supplementary material 1 (MP4 1052 KB)

## References

[CR1] Baum E, Peterson B, Böhm B, Dreizler A (2013). On the validation of LES applied to internal combustion engine flows: part 1: comprehensive experimental database. Flow Turbul Comb.

[CR2] Borée J, Maurel S, Bazile R (2002). Disruption of a compressed vortex. Phys Fluids.

[CR3] Borée J, Miles PC (2014). In-cylinder flow.

[CR4] Bücker I (2013) Experimental investigation of the in-cylinder flow of an internal combustion engine. PhD thesis, Aachen University, Aachen

[CR5] Bücker I, Karhoff D, Dannemann J, Pielhop K, Klaas M, Schröder W (2013). Comparison of PIV measured flow structures in two four-valve piston engines.

[CR6] Bücker I, Karhoff DC, Klaas M, Schröder W (2012). Stereoscopic multi-planar PIV measurements of in-cylinder tumbling flow. Exp Fluids.

[CR7] Celik I, Yanuz DC, Smirnov M (2001). Large eddy simulations of in-cylinder turbulence for internal combustion engines: a review. Int J Eng Res.

[CR8] Cosadia I, Borée J, Charnay G, Dumont P (2006). Cyclic variations of the swirling flow in a diesel transparent engine. Exp Fluids.

[CR9] Dannemann J, Pielhop K, Klaas M, Schröder W (2010). Cycle resolved multi-planar flow measurements in a four-valve combustion engine. Exp Fluids.

[CR10] Fetter DK, Suk E, Sullivan P (2000) PIV measurements within a water analog engine. Msc thesis, Toronto University, Toronto

[CR11] Freudenhammer D, Baum E, Peterson B, Böhm B, Jung B, Grundmann S (2014). Volumetric intake flow measurements of an IC engine using magnetic resonance velocimetry. Exp Fluids.

[CR12] Hess D, Tag S, Brücker C (2012) Volumetric flow studies in a 4-stroke water-analogue IC-engine using high-speed scanning-PIV. In: 16th International symposium on applications of laser techniques to fluid mechanics, Lisbon, 9–12 July 2012

[CR13] Heywood J (1987). Fluid motion within the cylinder of internal combustion engines—the 1986 Freeman scholar lecture. J Fluids Eng.

[CR14] Jayakumar C, Nargunde J, Sinha A, Henein NA, Bryzik W, Sattler E (2012). Effect of swirl and injection pressure on performance and emissions of JP-8 fueled high speed single cylinder diesel engine. J Eng Gas Turb Power.

[CR15] Khalighi B (1991). Study of the intake tumble motion by flow visualization and particle tracking velocimetry. Exp Fluids.

[CR16] Khalighi B, Huebler MS (1988) A transient water analog of a dual-intake-valve engine for intake flow visualization and full-field velocity measurements. In: SAE technical reports 880519, Warrendale, PA

[CR17] Koehler M, Hess D, Brücker C (2015). Flying PIV measurements in a 4-valve IC engine water analogue to characterize the near-wall flow evolution. Meas Sci Technol.

[CR18] Koehler M, Hess D, Kratzsch C, Brücker C (2014) Flying PIV measurements in a driven IC engine flow. In: 17th International symposium on application of laser techniques to fluid mechanics, Lisbon, Portugal, 7–10 July 2014

[CR19] Konrath R, Schröder W, Limberg W (2002). Holographic particle image velocimetry applied to the flow within the cylinder of a four-valve internal combustion engine. Exp Fluids.

[CR20] Lee KC, Yoo SC, Schock HJ (2000) Quantification of volumetric in-cylinder flow of SI engine using 3D laser doppler velocimetry. Seoul FISITA World Automotive Congress, 12–15 June, Seoul, Korea

[CR21] Lumley JL (1999). Engines-an introduction.

[CR22] Lumley JL (2001). Early work on fluid mechanics in the IC engine. Annu Rev Fluid Mech.

[CR23] Mahmood Z, Chen A, Yianneskis M, Ganti G (1996). On the structure of steady flow through dual-intake engine ports. Int J Numer Methods Fluids.

[CR24] Miles PC (2009). Turbulent flow structure in direct-injection, swirl-supported diesel engines.

[CR26] Pajkovic RV (2008). Spatial flow velocity distribution around an inlet port/valve annulus. Thermal Sci.

[CR27] Rabault J, Vernet JA, Lindgren B, Alfredsson PH (2016). A study using PIV of the intake flow in a diesel engine cylinder. Int J Heat Fluid Flow.

[CR28] Singh AP, Gupta A, Agarwal AK (2015) Tomographic particle image velocimetry for flow analysis in a single cylinder optical engine. SAE Int J Mater Manufact 8(2015–01-0599):472–481

[CR29] Söder M (2015) Creation and destruction of in-cylinder flows; Large eddy simulations of the intake and the compression strokes. PhD thesis, KTH Mechanics, Stockholm, Sweden (2015)

[CR30] Towers DP, Towers CE (2004). Cyclic variability measurements of in-cylinder engine flows using high-speed particle image velocimetry. Meas Sci Technol.

[CR25] van Overbrüggen T, Dannemann J, Klaas M, Schröder W (2013). Holographic particle image velocimetry measurements in a four-valve combustion engine. Exp Fluids.

